# Erratum to “Epidemiology and predictors of multimorbidity in Kharameh cohort study: a population‐based cross‐sectional study in southern Iran”

**DOI:** 10.1002/hsr2.1025

**Published:** 2022-12-24

Moftakhar L, Rezaeianzadeh R, Ghoddusi Johari M, Hosseini SV, Rezaianzadeh A. Epidemiology and predictors of multimorbidity in Kharameh cohort study: a population‐based cross‐sectional study in southern Iran. *Health Sci Rep*. 2022;e988. doi:10.1002/hsr2.988

In the article by Moftakhar et al., the authors' information was incorrect in the author byline.

The correct authors' names and affiliations are as follows:

Masoumeh Ghoddusi Johari

Breast Diseases Research Center, Shiraz University of Medical Sciences, Shiraz, Iran.

Seyed Vahid Hosseini

Colorectal Research Center, Shiraz University of Medical Sciences, Shiraz, Iran.

We apologize for the error.

